# Elucidation of terpenoid metabolism in *Scoparia dulcis* by RNA-seq analysis

**DOI:** 10.1038/srep43311

**Published:** 2017-03-07

**Authors:** Yoshimi Yamamura, Fumiya Kurosaki, Jung-Bum Lee

**Affiliations:** 1 Graduate School of Medicine and Pharmaceutical Sciences for Research, University of Toyama, 2630 Sugitani, Toyama, Toyama 930-0194, Japan

## Abstract

*Scoparia dulcis* biosynthesize bioactive diterpenes, such as scopadulcic acid B (SDB), which are known for their unique molecular skeleton. Although the biosynthesis of bioactive diterpenes is catalyzed by a sequence of class II and class I diterpene synthases (diTPSs), the mechanisms underlying this process are yet to be fully identified. To elucidate these biosynthetic machinery, we performed a high-throughput RNA-seq analysis, and *de novo* assembly of clean reads revealed 46,332 unique transcripts and 40,503 two unigenes. We found diTPSs genes including a putative *syn*-copalyl diphosphate synthase (SdCPS2) and two kaurene synthase-like (SdKSLs) genes. Besides them, total 79 full-length of cytochrome P450 (CYP450) genes were also discovered. The expression analyses showed selected CYP450s associated with their expression pattern of *SdCPS2* and *SdKSL1*, suggesting that CYP450 candidates involved diterpene modification*. SdCPS2* represents the first predicted gene to produce *syn*-copalyl diphosphate in dicots. In addition, *SdKSL1* potentially contributes to the SDB biosynthetic pathway. Therefore, these identified genes associated with diterpene biosynthesis lead to the development of genetic engineering focus on diterpene metabolism in *S. dulcis.*

Plants produce a diverse array of terpenes, the largest class of plant-derived natural products. They range from simple flavor and fragrance compounds, such as limonene, to complex triterpenes, and have numerous potential applications across the food and beverage, pharmaceutical, cosmetic, and agriculture industries. However, there are limitations to the use of large amounts of these compounds as they are naturally produced in small quantities and intricate steps are required for extraction and/or purification. In addition, their chemical syntheses are inherently difficult under stereochemical control due to their complex structure.

Large populations of terpenes are cyclic compounds possessing several chiral centers, and the cyclization catalyzed by terpene synthases (TPSs) is the first step to create diverse structures of terpenes. Therefore, discovery and elucidation of TPSs may provide important information about terpene synthesis in plants. In addition, this information could be used to construct metabolic engineering machinery to produce large quantities of terpenoids under stereochemical control in a tractable microorganism^1^,^2^,^3^.

Sweet broomweed (*Scoparia dulcis* L.) is a perennial herb widely distributed in the torrid zone, and has been recently placed in the Plantaginaceae family (formally Scrophulariaceae)^4^. In these districts, plants have been used as a medication for stomach disorders, diabetes, hypertension, bronchitis, and insect bites^5^, and clinical trial of *S. dulcis* leaf extract has recently performed in Sri Lanka^6^. Phytochemical studies revealed that this plant produce various unique diterpenes its leaves: (1) labdane type: scoparic acid A; (2) scopadulane type: scopadulcic acid B (SDB) and scopadulciol; (3) aphidicolane type: scopadulin^7^. Among these diterpenes, SDB was found to possess various biological activities such as antiherpetic and inhibitory effects on gastric H^+^, K^+^ -ATPase^8^,^9^. In addition to these diterpenes, miscellaneous biologically active diterpenes have also been isolated from *S. dulcis*^10^,^11^,^12^,^13^. Furthermore, numerous triterpenoids have also been isolated from *S. dulcis* as bioactive substances^14^. Thus, *S. dulcis* might be important medicinal resources for providing unique bioactive terpenoids. Due to the unique carbon skeleton and biological activities of SDB, the scopadulane type diterpenes were selected as attractive targets for chemical synthesis and their total syntheses were accomplished by several groups^15^,^16^,^17^. However, their synthetic route included numerous steps and produced SDB as racemic mixtures.

When we evaluated the metabolites produced by *S. dulcis* from a different perspective, it was clear that *S. dulcis* may harbor unique biosynthetic enzymes to produce terpenoids. The putative biosynthetic machinery of the unique diterpenes, in particular SDB, can be divided into four stages: (1) synthesis of isopentenyl diphosphate (IPP) and its isomer dimethylallyl diphosphate (DMAPP); (2) conversion of diterpene precursors, geranylgeranyl diphosphate (GGPP), from IPP and DMAPP; (3) cyclization of GGPP to *syn-*copalyl diphosphate (*syn*-CPP), then a second cyclization to produce intermediate species possessing scopadulane skeleton; (4) redox modification and esterification by CYP450s and benzoyl CoA transferase, respectively. In the first stage, there are two biosynthetic routes, the 2-*C*-methyl-D-erythritol 4-phosphate (MEP) pathway and the mevalonic acid (MEV) pathway, and SDB has been shown to be produced *via* the MEP pathway by [^13^C]-glucose and inhibitor feeding experiments^18^,^19^. In the second stage, two GGPP synthases (GGPPSs) from *S. dulcis* have been cloned and functionally characterized^20^,^21^. In addition, our previous study revealed that *SdGGPS1* and *SdGGPS2* were expressed as constitutive and inducible homologous genes, respectively^22^. However, the enzymes involved in cyclization and modification to produce SDB at the third and fourth stages described above are still unknown, whereas *ent*-copalyl diphosphate synthase (SdCPS1) has been cloned and functionally characterized^23^.

Recent progress in next-generation sequencing techniques has facilitated the discovery of novel gene candidates in non-model organisms. This progress prompted us to carry out transcriptome analysis to discover gene candidates and to elucidate the mechanisms of terpenoid metabolism of *S. dulcis* using Illumina RNA-Seq technology. Here we report transcriptome analysis of different organs of *S. dulcis* in response to methyl jasmonate treatment. Combined with quantitative RT-PCR and phylogenetic analysis, our results showed novel aspects of terpenoid metabolism in *S. dulcis*.

## Results

### De novo assembly of the *S. dulcis* transcriptome

To establish a transcriptome catalogue of *S. dulcis*, we used MiSeq pair-end technology for sequencing transcriptome, which enabled us to create an assembly of contigs. To achieve this, we prepared four cDNA libraries from three tissues including leaves with or without MeJA treatment, young leaves, and roots. We choose these samples for preparation of total RNA, since it is also known that SDB production in *S. dulcis* leaf tissue is rapidly and transiently stimulated by MeJA as an elicitor^24^, and SDB contents in young leaves were higher than those in adult leaves^25^. In total, there were approximately 20.6 million raw reads from *S. dulcis* (Supplementary Table S1). The sequencing raw data have been submitted to the DDBJ Sequence Reads Archive (DRA) under the accession number DRA004058. Quality trimming and filtration resulted in 20.5 million cleaned reads that were assembled using Trinity, and generated 60,012 transcripts with an average length of 938 bp and an N50 of 1,430 bp. The sequences were clustered using the CD-HIT-EST to remove any redundant sequences. After clustering of the sequences with 95% identity, 46,332 transcripts with an average length of 934 and an N50 of 1,464 bp were generated (Table 1).

### Functional annotations of transcripts

To make a functional annotation and classification of the putative identities of the assembly, all unigenes were searched against public databases including the non-redundant protein (Nr), non-redundant nucleotide sequence (Nt), Uniprot/Swiss-Prot, and Cluster of Orthologous Groups of proteins (COG). The best hits were selected from the matches with an E-value of less than 10^−5^.

30,471 (65.8%), 17,663 (38.1%), 22,485 (48.5%), and 10,105 (21.8%) unigenes were annotated based on BLASTx (cut-off E-value 10^−5^) searches of the public databases; Nr, Nt, Swiss-Prot, and COG, respectively (Supplementary Figure S1). In total, 30,872 annotated sequences were identified, as shown in Supplementary Table S2. Among these annotated unigenes, the species with the highest number of best hits were sesame (*Sesamum indicum*, 65.5% matched gene) and common monkey-flower (*Erythranthe guttata*, formerly *Mimulus guttatus*, 13.6% matched gene) (Supplementary Table S3). These findings are consistent because sesame and common monkey-flower species both belong to Lamiales with sequenced genomes.

**Figure 1:**
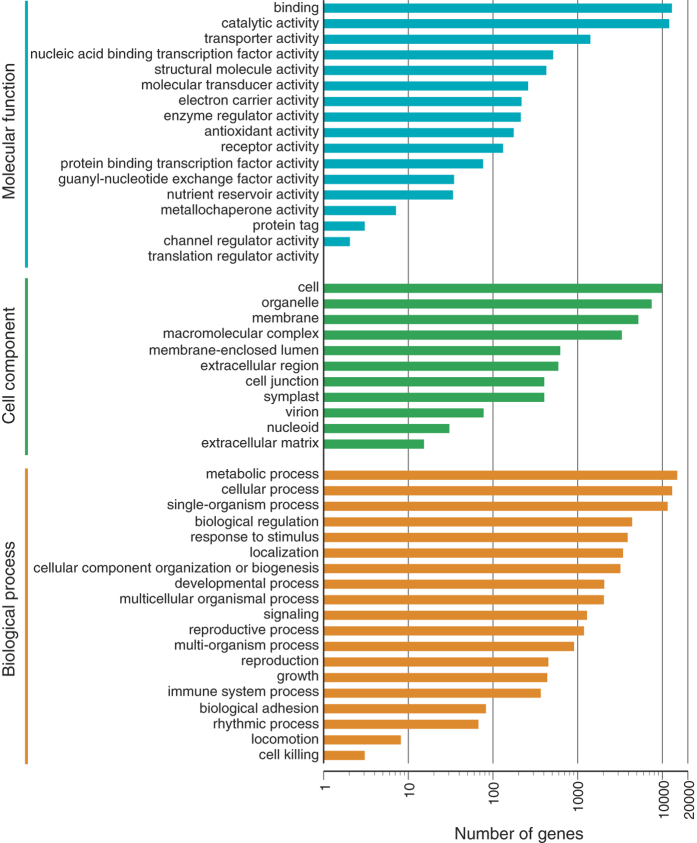
Histogram of GO classifications of assembled *Scoparia dulcis* unigenes. The results are grouped into three main categories: biological process, cellular component, and molecular function.

Gene Ontology (GO) terms were subsequently assigned to *S. dulcis* unigenes based on their sequence matches to known protein sequences using the Blast2GO program with Nr annotation. Unigenes were classified into 47 groups that could be categorized into three main classifications: “biological process”, “cellular component”, and “molecular function” (Fig. 1). In the biological process category, cellular process (12,804 unigenes) and metabolic process (14,704 unigenes) represented the major contributors. In the molecular function category, binding (12,720 unigenes) and catalytic activity (11,834 unigenes) represented the major contributors. In addition, gene classification in the “metabolic process” was shown in Supplementary Figure S2.

Functional classification and pathway assignment were also performed using the Kyoto Encyclopedia of Genes and Genomes (KEGG). In total, 142 KEGG pathways, including 10,328 unigenes, were found in this study (Supplementary Table S2). Among them, 381 unigenes were involved in the biosynthesis of secondary metabolites, of which 156 were for terpenoids, 103 for phenylpropanoids, 25 for flavonoids, 35 for alkaloids, and 34 for other metabolites.

### Prediction of genes involving terpenoids biosynthesis

**Figure 2:**
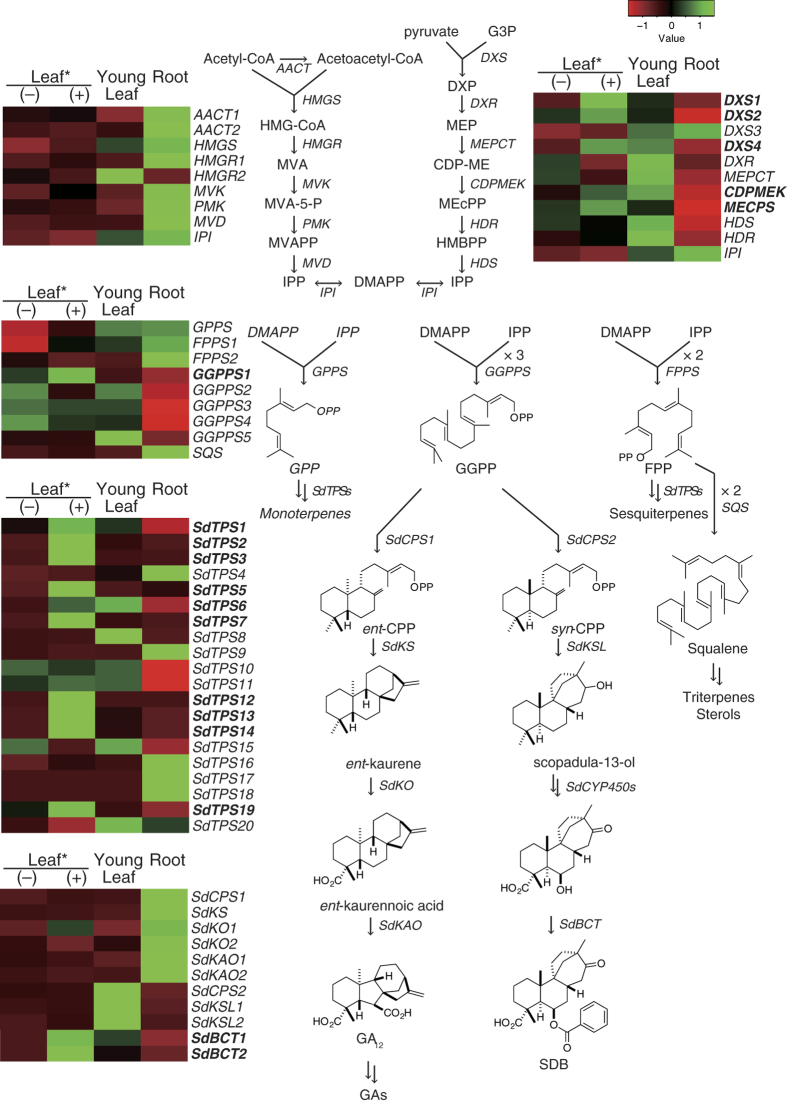
Prediction of terpenoid metabolic pathways and differentially expressed orthologous genes in *Scoparia dulcis*. Heatmap depicting the expression profile of isoprenoid and terpene metabolism-related genes in young leaf, leaf (+) with or (−) without treatment of MeJA, and root tissues of *S. dulcis*. MeJA-inducible genes were shown in bold face. Color gradient illustrating the Z-score of the gene expression values by calculating the FPKM values.

To investigate terpenoid metabolism in *S. dulcis*, we conducted tissue-specific expression analysis of candidate unigenes responsible for terpenoid biosynthesis. Combined with the data obtained using the Blast2Go software, BLAST (tblastn) and HMMER approaches were also used to predict the candidates. In the tblastn search, we selected corresponding protein sequences from *Arabidopsis thaliana* and *Oryza sativa*, and searched against an *in-house* transcripts database of *S. dulcis*. In the present study, we detected gene candidates involved in MEV and MEP pathways, which are biosynthetic pathways providing general isoprenoid precursors, isopentenyl diphosphate (IPP), and dimethylallyl diphosphate (DMAPP). As shown in Fig. 2, almost all of the gene candidates involved in the MEV pathway were predominantly expressed in root tissue, whereas those involved in the MEP pathway were in leaves, in particular young leaf tissue. In addition, distinctive expression patterns were observed in some of the genes. 3-Hydroxy-3-methylglutaryl-CoA reductase 1 (*HMGR1*) was specifically expressed in root tissue, whereas *HMGR2* were in young leaf tissue. Moreover, of the four 1-deoxy-D-xylulose 5-phosphate synthase (*DXS*) genes analyzed, *DXS1* and *DXS2*, which were induced by MeJA, were expressed in leaf tissues, whereas *DXS3*, which was not induced by MeJA, was specifically expressed in root and young leaf tissues. In addition, the expression pattern of *DXS4* was different from that of other *DXSs; DXS4* was expressed predominantly in young leaf tissue and was induced by MeJA stimulation.

Then, we identified and examined the expression profiles of genes involved in terpenoid precursor biosynthesis such as geranyl diphosphate synthase (*GPPS*), farnesyl diphosphate synthase (*FPPS*), geranylgeranyl diphosphate synthase (*GGPPS*), and squalene synthase (*SQS*). As shown in Fig. 2, *FPPSs* and *SQS* were predominantly expressed in root tissue, whereas *GPPS* was expressed in both young leaf and root tissue. On the other hand, *GGPPSs* were abundantly expressed in leaves when compared with expression in roots. In addition, the present result is consistent with previous data that *GGPPSs* might be composed of several homologous genes^22^.

Next, we attempted to extract gene candidates responsible for the formation of terpene skeletons in *S. dulcis* using a HMMER search of translated amino acid sequences of transcripts in a Pfam database. From our RNA-seq data, 26 unigenes contained conserved domains [terpene synthase N terminal domain (PF01397) and terpene synthase metal binding domain (PF03936)], as summarized in Table. 3. Among them, 20 genes (*SdTPS1 to 20*) were suggested to be mono- and sesquiterpene synthase genes based on the sequence homology to functionally characterized TPSs. As shown in Fig. 2, some *SdTPS*s were induced by MeJA, whereas five *SdTPS*s, *SdTPS4, SdTPS9, SdTPS16, SdTPS17, and SdTPS18*, were suggested to express constitutively in roots. On the other hand, the other five genes were suggested to be involved in diterpene biosynthesis, and two genes were identical with previously isolated genes, *ent-*copalyl diphosphate synthase (*SdCPS1*) and kaurene synthase (*SdKS*). The remaining three homologous gene candidates were also predicted and named as putative *syn-CPS (SdCPS2*) and kaurene synthase-like genes (*SdKSLs*). SdKSL1 shows almost full-length cyclase (804 amino acids in length), however, the longest ORF of the SdKSL2 sequence was not long enough to encode a class I terpene cyclase (only 519 amino acids in length). This indicates that only SdKSL1 can be reliable categorized as a class I diterpene cyclase.

The biosynthetic route of SDB has been predicted, as illustrated in Fig. 2. Previous studies have indicated that SDB is accumulated in the aerial part of the plant, in particular in young leaves^25^, and that the biosynthesis of SDB might be dependent on differentiation of leaves^26^. It has been suggested that the biosynthetic gene for SDB may be expressed in leaf tissue, therefore, we identified candidate genes involved in diterpene metabolism in *S. dulcis* on the basis of their expression patterns. In contrast to *SdTPS* genes, expression of diterpene synthase genes were easily clarified in a tissue-specific manner. *SdCPS1* and *SdKS* were specifically expressed in roots, whereas *SdCPS2* and *SdKSLs* were expressed in young leaves. In addition, *SdKO1, SdKO2,* and *SdKAO1*, which are involved in gibberellin biosynthesis, were also predominantly expressed in root tissues. Therefore, it was suggested that gibberellin biosynthesis was active in root tissue, probably in the meristem, at the stage when transcripts were obtained (8-week old plants).

**Figure 3:**
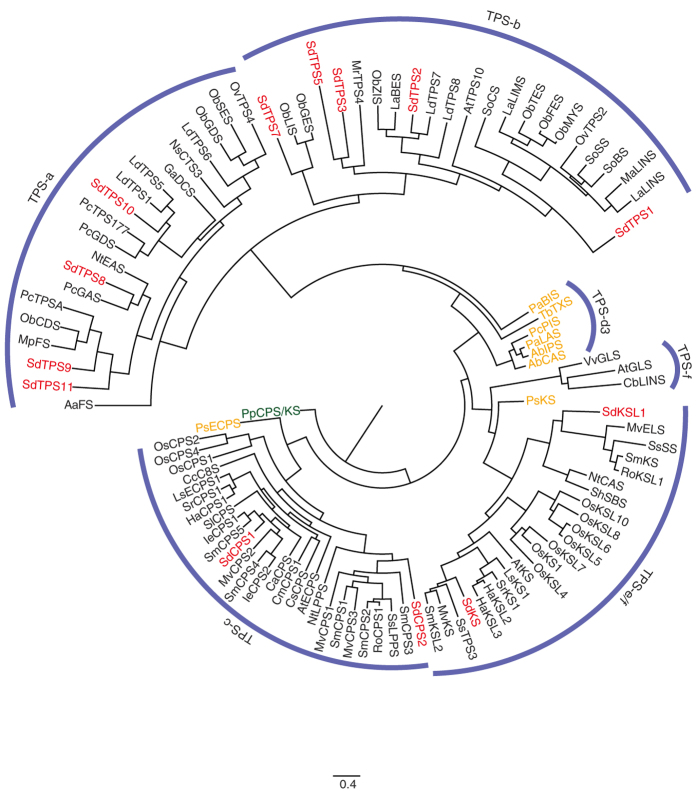
Phylogenetic analyses of TPSs from *S. dulcis*. The maximum likelihood tree illustrates the phylogenetic relatedness of *S. dulcis* terpene synthases of other species of the terpene synthases. The ancestral *Physcomitrella patens ent*-kaurene/kaurenol synthase was used to root the tree. Descriptions of terpene synthases used in the phylogeny are listed in Table 3 and Supplementary Table S4. Red- and tangeline-marked enzymes show terpene synthases from *S. dulcis* and gymnosperms, respectively.

It has been reported that phylogenetic analyses of TPS protein sequences recognized seven major clades, and the function and distribution of plant TPS subfamilies have been summarized^27^. Thus, phylogenetic comparison of the translated sequences of *TPSs* might help to predict their function. As shown in Fig. 3, we applied nine TPSs, which contained full-length of ORFs, to phylogenetic analyses with known TPSs, summarized in Supplementary Table S4, and categorized them into appropriate clades. SdTPS8, SdTPS9, SdTPS10, and SdTPS11 were placed into the TPS-a subfamily, which is reported to be involved in sesquiterpene synthesis. SdTPS10 was closely related to LdTPS1 (δ-cadinene synthase) and LdTPS5 (bicyclogermacrene synthase), and SdTPS8 was closely related to PcGAS (germacrene A synthase) and NtEAS (*epi*-aristrochene synthase). SdTPS9 and SdTPS11 showed close phylogenetic relationships with PcTPSA (γ-curcumene synthase), ObCDS (γ-cadinene synthase), and MpFS (β-farnesene synthase). Therefore, these four SdTPSs were suggested to be involved in sesquiterpene biosynthesis in *S. dulcis.*

SdTPS1, SdTPS2, SdTPS3, SdTPS5, and SdTPS7 were assigned to the TPS-b subfamily, which predominantly contains monoterpene synthases from angiosperms. Among them, SdTPS1 was placed into a sub-clade consisting of monoterpene cyclases, and SdTPS7 was closely related to acyclic oxygenated monoterpene synthases such as ObGES (geraniol synthase) and ObLIS (*R*-linalool synthase). SdTPS3 and SdTPS5 showed a close relationship with MrTPS4 (β-ocimene synthase). Therefore, these four terpene synthases were suggested to be involved in monoterpene metabolism. On the other hand, SdTPS2 belonged to the sub-clade consisting of monocyclic sesquiterpene synthases such as ObZIS (α-zingiberene synthase) and LdTPS7 (trans-α-bergamontene synthase). This data suggests that SdTPS2 might be a monocyclic sesquiterpene synthase.

SdCPS1 and SdCPS2 were placed into the TPS-c clade, whereas SdKS and SdKSL1 were placed into the TPS-e/f clade. The TPS-c and TPS-e/f clades contain exclusively monofunctional class II and class I enzymes, respectively. SdCPS1 has previously been functionally annotated as an *ent*-CPS^23^, and this enzyme showed close relationships with other *ent-*CPSs from Lamiales. On the other hand, SdCPS2 was placed into a sub-clade distinct from those of *ent-*CPSs, and it was closer related to diTPSs involved in specialized metabolism, such as oxygenating diterpene synthases (SsLPPS, labda-13-en-8-ol diphosphate synthase) and (+)-CPSs like MvCPS3 and SmCPS1. In addition, sequence alignment also revealed our SdCPS2 could be distinguished from *ent*- and (+)-CPSs (Supplementary Figure S4). Potter *et al*. has reported that H263 and N322 residues are key catalytic base dyads in *A. thaliana*^28^, and that they are well conserved in *ent*-CPSs. In the case of SmCPS1, the corresponding residues were F256 and H315, and these are well conserved in (+)-CPSs. However, sequence alignment showed that the corresponding residues of SdCPS2 were F279 and P340, and that they did not agree with those in *ent*- and (+)-CPSs. Therefore, SdCPS2 was suggested to be different from the enzymatic activity of *ent-* and (+)-CPSs.

Phylogenetic analysis also suggested that two of the class I diTPSs (SdKS and SdKSL1) may be functionally distinct since SdKS and SdKSL1 showed close relationships with *ent*-kaurene synthases and known diTPSs with specialized functions such as MvELS (9,13-epoxy-labd-14-ene synthase) and SsSS (sclareol synthase). Thus, SdKSL1 was deduced to catalyze the cyclization step of *syn-*CPP in the pathway of unique and specialized diterpene metabolism in *S. dulcis*.

Finally, SDB and other unique diterpenes produced by *S. dulcis* are substituted with a benzoyl unit at the C-6 position. Thus, it was suggested that benzoyl-CoA transferase (BCT) also plays an important role in diterpene metabolism in *S. dulcis*. To predict the responsible gene(s), benzoyl-CoA:taxane 2α-*O*-benzoyltransferase from *Taxus cuspidata* (AF297618) was used to search against an *in-house* transcriptome database using the TBLASTN approach. As a result, two putative candidates, *SdBCT1* and *SdBCT2*, were obtained that were expressed in young leaf tissue. In addition, they were found to be induced by MeJA, as shown in Fig. 2. Thus, these genes were suggested to be involved in unique diterpene biosynthesis in *S. dulcis*.

### Prediction and classification of CYP450 genes in *S. dulcis*

Terpene diversification is driven by the machinery consisting of TPSs and cytochrome P450-dependent monooxygenases (CYP450s). The latter is important for modifying and diversifying the terpenoid scaffolds by redox modification. Therefore, we examined the CYP450s responsible for the terpenoid biosynthesis in *S. dulcis*. By searching for transcripts possessing the cytochrome p450 domain (PF00067) against a Pfam-A database, 341 candidates were detected. After detecting ORFs, we found 87 full-length CYP450 genes in *S. dulcis.* In addition, four CYP gene fragments, which were identical with those previously isolated from *S. dulcis*, were also added to the candidates. Subsequently, those CYP450 ORFs was classified by comparison with amino acid sequences derived from typical plant CYP450s. As shown in Table 2, the amino acid length of CYP450s ranged from 403 to 544, and most of them (62/87) were suggested to be present in a secretory pathway, *i.e.,* they were inserted into the ER membrane, since they contained signal peptides.

**Figure 4:**
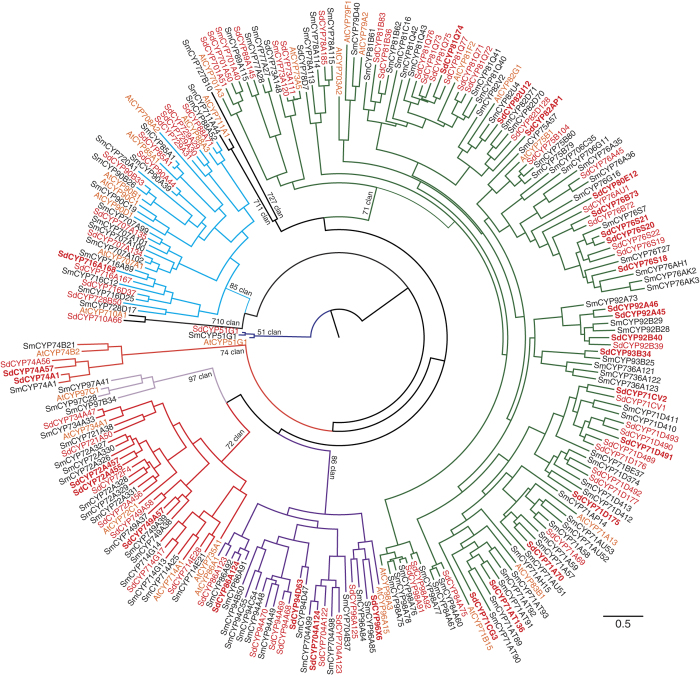
Phylogenetic analyses of CYP450s from *S. dulcis, S. miltiorrhiza*, and *A. thaliana*. The unrooted maximum likelihood tree illustrates the phylogenetic relatedness of CYP450s from *S. dulcis* (red), *Salvia miltiorrhiza* (black), and *A thaliana* (tangeline). Descriptions of CYP450s are listed in Table 2, Supplementary Table S5, and Supplementary Table S6, respectively.

Then, we comparative analyzed SdCYPs against *S. miltiorrhiza*. The sequences of 119 CYP450 proteins (SmCYPs) and 91 SdCYPs were used to construct trees for CYP450s by maximum likelihood estimation (Fig. 4). As a result, 52 SdCYP450s were A-type and distributed into 12 families, whereas 39 were non-A type and belonged to 16 families and 7 clans. Among them, genes belonging to the CYP71 clan have been reported to be involved in secondary metabolism^29^,^30^. Moreover, TPS genes were predominantly found in combination with CYP71 clan genes, such as CYP71, CYP76, and CYP99 families, in angiosperms^31^,^32^,^33^. CYP71D16 from a tobacco plant and CYP71D51 from a tomato plant have been reported to catalyze hydroxylation of cembrenediol and lycosantalene, respectively^33^,^34^. CYP76 members in rice have been shown to be involved in diterpene metabolism^35^,^36^,^37^,^38^,^39^. In addition, CYP76AH and CYP76AK sub-family members are responsible for diterpene hydroxylation in Lamiales^40^,^41^,^42^,^43^,^44^. In the present study, fourteen CYP71 and nine CYP76 genes were obtained from *S. dulcis* (Table 2). Among the CYP71 family genes, CYP71D and CYP71CV genes were assigned to same clan, as shown in Fig. 4. Therefore, CYP71CV, CYP71D, and CYP76 families were suggested to be candidates involved in unique diterpene metabolism in *S. dulcis*.

On the other hand, CYP716 and CYP51 family genes might be involved in triterpene biosynthesis^31^,^45^,^46^. As shown in Fig. 4, three genes and one gene were found to belong to the CYP716 and CYP51 families, respectively. So far, it has been reported that *S. dulcis* produces several triterpenes, such as a betulinic acid, therefore, these CYPs may be involved in triterpene metabolism.

### Real-time PCR analysis of putative genes involved in diterpene biosynthesis

**Figure 5:**
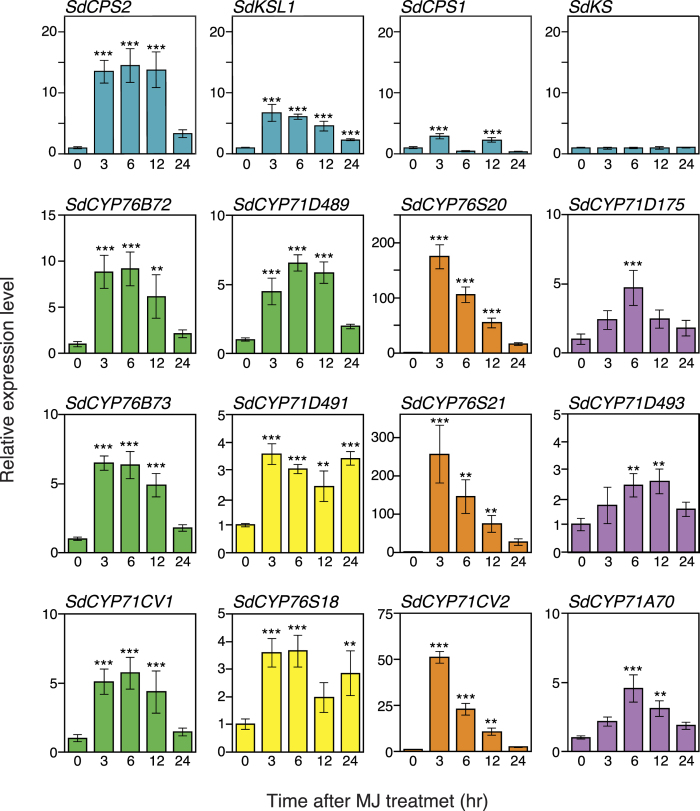
qRT-PCR analysis of the expression level of diTPSs and selected SdCYPs by MeJA treatment. Leaves were harvested at indicated time points after treatment with 0.1 mM MeJA. 18S rRNA gene was used for normalization. The transcript levels of each gene in the leaf at 0 hr were set to 1.0. CYPs were grouped into four patterns (green, yellow, orange, and purple) based on their expression patterns. Data are shown as mean ± SD (n = 3). Asterisks indicate significant differences from the control (*p < 0.05, **p < 0.01, and ***p < 0.001).

As described above, we discovered novel candidate genes involved in the biosynthesis of unique diterpenes in *S. dulcis*. To clearly elucidate their function, we examined their expression levels when stimulated by MeJA. As shown in Fig. 5, *SdCPS2* and *SdKSL1* were immediately induced by MeJA stimulation and their strong inductions were continued until 12 h post administration, whereas the expression levels of *SdCPS1* and *SdKS* differed from those of *SdCPS2* and *SdKSL1*. It was noteworthy that MeJA treatment did not alter the relative expression level of *SdKS.*

To detect *SdCYP*s induced coordinately with *SdCPS2* and *SdKSL1*, we selected twelve genes based on the data of their expression patterns in tissues, as shown in Supplementary Figure S3. Several SdCYPs belonging to the CYP71 and CYP76 families were analyzed following treatment with MeJA. As shown in Fig. 5, expression patterns of *SdCYP*s could be classified into four patterns. Four *SdCYPs* (shown with green bars), such as *SdCYP71CV1, SdCYP71D489, SdCYP76B72, and SdCYP76B73*, were up-regulated at 3 h post-treatment with MeJA. It was noteworthy that their expression patterns were consistent with those of *SdCPS2* and *SdKSL1*, therefore, they were considered to express coordinately with these *TPS*s. On the other hand, expression levels of *SdCYP76S20, SdCYP76S21*, and *SdCYP71CV2* (shown with orange bars), were increased *ca* 175-fold, *ca* 250-fold, and *ca* 50-fold, respectively, compared with those before MeJA treatment, and appeared to be induced strongly and transiently by stimuli. However, these expression patterns were quite different from those of *SdCPS2* and *SdKSL1*. Expression of *SdCYP71D175, SdCYP71D493, and SdCYP71A70* (shown with purple bars), increased slowly and reached maximum expression at 6 to 12 h post-treatment with MeJA. In addition, expression patterns of *SdCYP71D491* and *SdCYP76S18* (shown with yellow bars), showed a bimodal pattern.

## Discussion

Recent progress in next-generation sequencing technologies has expanded the capabilities for studying non-model plants. Therefore, we utilized these methodologies to sequence the transcriptome in the present study, and identified a large number of novel genes in *S. dulcis*. Consequently, we could postulate the mechanisms of terpenoid metabolism in *S. dulcis* by identification of gene candidates for terpene biosynthesis. Recently, interest in the biosynthesis of terpenes, in particular diterpenes, has gradually increased due to their industrial and scientific importance. Therefore, our present study provides important information for plant sciences and/or natural products chemistry.

The Lamiales include a large number of economically important plants, and most of them produce a huge number of terpenes. Several Lamiales have been used as medicinal plants, and their bioactive principles are unique diterpenes. For example, *Isodon* plants produce a large array of *ent*-kaurene-type diterpenes^47^ and *S. miltiorrhiza* biosynthesize a miltiradiene that is an intermediate of tanshinone biosynthesis^48^. When considering the biosynthetic machineries of these diterpenes, careful attention must be paid to their stereochemistry. Briefly, the former *Isodon* diterpenes are synthesized via *ent*-CPP by *ent*-CPS, whereas the latter, miltiradiene, is biosynthesized via (+)-CPP by (+)-CPS. Therefore, these enzymes might be important for diversification of diterpenes in nature. Indeed, it is suggested that SDB might be synthesized via *syn*-CPP in *S. dulcis* because of its stereochemical configuration. To date, *syn*-CPS has been solely isolated from *O. sativa*^49^,^50^, and the rice *syn*-CPS (OsCPS4) has been implicated in the biosynthesis of phytoalexins, momilactones, and oryzalexin S. Despite the rice *syn*-CPS being well studied, a *syn*-CPS has not yet been identified from dicots because few diterpenes are synthesized via *syn*-CPP. When we phylogenetically analyzed TPSs being predicted by *de novo* assembly of transcripts, SdCPS2 was placed into a sub-clade consisting of (+)-CPSs and oxygenating diterpene synthases, which was relatively far from a sub-clade consisting of rice CPSs (Fig. 3). Furthermore, amino acid sequence alignment also revealed distinct properties in important catalytic base amino acid residues in the rice *syn*-CPS, OsCPS4^51^ (Supplementary Figure S4). OsCPS4 contains H251 and C310 residues at the same position proposed to be the catalytic base dyad in *ent-*CPS, however, alanine substitution did not significantly alter their activity. In a previous report, Potter *et al*. showed that a H501 residue presented in the active cavity is an important catalytic base to produce *syn*-CPP^51^. In our SdCPS2, Y528 corresponded to H501 of OsCPS4, and tyrosine is invariably conserved in plant CPS. Thus, the control of stereochemically unique reactions is suggested to be different in SdCPS2 from that in OsCPS4, although the enzymatic reaction is hypothesized to be the same. We are now currently focusing on characterizing/elucidating the enzymatic reaction of SdCPS2. Similarly, SdKSL1 was also deduced to be involved in specialized diterpene metabolism, as described above, since it showed close relationships with MvELS and SsSS.

Frequently, genes associated with identical metabolic pathways are often co-expressed so that they can catalyze a linear chain of reactions^52^. In the present study, we used a criteria based on differential expression patterns and qPCR analyses to choose gene candidates involved in diterpene metabolism. We found distinct patterns between *SdCPS1-SdKS* and *SdCPS2-SdKSL1* linages (Fig. 5). So far, it has been shown that SDB synthesis is significantly induced by exposure to MeJA^24^. Putative *SdCPS2* and *SdKSL1* were induced at 3 h post-treatment with MeJA and their expression persisted even at 12 h post-treatment. In addition, we selected four CYP450 candidates, *SdCYP71CV1, SdCYP71D489, SdCYP76B71*, and *SdCYP76B72*, involved in SDB biosynthesis. As shown in Fig. 2, it was suggested that three CYPs might be involved in SDB biosynthesis, such as in the hydroxylation of C-6 and carboxylation of C-18. Therefore, these CYP genes might be the most likely candidates for redox modification of diterpene precursor in *S. dulcis*.

While SdCPS2 has not yet been characterized, it seems likely to produce the *syn*-CPP intermediate required for SDB biosynthesis, which would provide the first example of such a *syn*- specific CPS from dicots. Further functional investigations of SdCPS2 and SdKSL1 have already begun, and the results will be published in the near future. The transcriptome sequences and gene expression profiles provide a solid foundation for functional genomic studies of *S. dulcis* in the future and will facilitate a better understanding of the molecular mechanisms of diterpenes (SDB) biosynthesis.

## Conclusion

The present paper revealed that transcriptome analyses provide useful information about novel gene discovery. We revealed gene candidates involved in terpene metabolism in *S. dulcis*. Among the identified genes, *SdCPS2* represents the first gene to produce *syn*-copalyl diphosphate in dicots. In addition, *SdKSL1* was also suggested to participate in the SDB biosynthetic pathway. In addition to these two genes, other candidate genes involved in SDB biosynthesis, were also identified from the results of our RNA-seq analysis. qPCR analyses provided evidence that CYP450s participated in diterpene metabolism. Therefore, these identified genes associated with diterpene biosynthesis will facilitate research and genetic engineering of diterpene metabolism in *S. dulcis.*

## Methods

### Plant material and MeJA treatment

*S. dulcis* were grown under sterilized conditions in 1/2 Murashige and Skoog (MS) agar media under constant light conditions at 25 °C. Eight-week-old plants were used for MeJA treatment.

For RNA-seq library, the plants were treated with or without 0.1 mM MeJA (Sigma-Aldrich, MO, USA) using sprays, and leaves were harvested after 24 h. At this time point, we confirmed the enhanced production of SDB by HPLC using a previously established method^24^. Various tissues, such as young leaves (first and second leaf set from the top), mature leaves (third leaf set from the top treated with or without MeJA), and roots were harvested and frozen immediately in liquid nitrogen, and stored at −80 °C for RNA extraction. For qRT-PCR, plants were kept for 0, 3, 6, 12, and 24 h at 25 °C after MeJA treatment. At each time point, samples were collected from three or four separated plants and directly frozen in liquid nitrogen.

### RNA-seq library construction

Total RNAs were isolated using a TRIzol reagent (Invitrogen, CA, USA). The integrity of total RNA was checked using Agilent 2100 Bioanalyzer. The mRNA was isolated from total RNA using PolyATtract^®^ mRNA Isolation Systems (Promega, MA, USA), and the RNA-seq libraries were constructed using the SMARTer^®^ stranded RNA-Seq kit (Clontech, CA, USA). The library was sequenced using an Illumina MiSeq sequencer (Illumina, CA, USA) after checking the quality with an Agilent 2100 Bioanalyzer.

### Data processing, assembly and annotation

The raw reads were cleaned by removing reads containing adapter, reads containing poly-N, and low quality reads using FASTX Toolkit (http://hannonlab.cshl.edu/fastx_toolkit/) and PRINSEQ^53^. Sequence quality was examined using FastQC (http://www.bioinformatics.babraham.ac.uk/projects/fastqc). *De novo* assembly of clean reads was performed using Trinity^54^. The resulting *de novo* assembly was clustered using CD-HIT with 95% global sequence identity^55^.

All the assembled unigenes were searched against the Nr database to identify the putative mRNA functions using an E-value cut-off of 10^−5^. Functional annotation and Gene Ontology analysis was carried out using Blast2Go software^56^.

### Abundance estimation and differential expression analysis

Gene expression analysis was carried out with RSEM^57^ bundled with the Trinity package. Differentially expressed transcripts across the tissues were identified and clustered according to expression profiles using EdgeR Bioconductor package^58^ using R statistical software.

### Computational prediction of TPS and CYP450 genes in *S. dulcis*

Computational prediction of TPS and CYP450 genes were performed under the following criteria. Coding regions of transcripts were extracted using Transdecoder, and were searched by HMMER against the Pfam-A database with an E-value cutoff of 1e-5. The ORFs matching the HMM model (PF00067, or PF01397 and PF03936) were selected as CYP450 or TPS candidates, respectively. The hit candidate genes were then searched against the CYPED database^59^ and SwissProt database with an E-value cutoff of 1e-5.

To perform phylogenetic analysis, multiple sequence alignments were performed on the TPS or CYP homologs. The MAFFT program was used in these alignments by employing a highly accurate method: L-INS-I^60^. Maximum likelihood (ML) trees were built on the datasets using RAxML^61^. RAxML analyses were conducted with the JTT model and 500 replicates of bootstrap analyses, and the obtained phylogeny was displayed using FigTree (http://tree.bio.ed.ac.uk/software/figtree).

### qPCR analysis of selected candidate genes responsible for diterpene biosynthesis

First-strand cDNAs were synthesized using a PrimeScript™ II 1st strand cDNA Synthesis Kit (Takara Bio Inc., Shiga, Japan). The resulting first-strand cDNAs were used as templates for qPCR. Real-time PCR was performed using Brilliant III Ultra-Fast SYBR^®^ Green QPCR Master Mix on an Mx3005p real-time QPCR system (Agilent Technologies). *S. dulcis* 18S rRNA gene (JF718778) was used for normalization. The sequences of primers used in this study are listed in Supplemental Table S7.

## Additional Information

**How to cite this article**: Yamamura, Y. *et al*. Elucidation of terpenoid metabolism in *Scoparia dulcis* by RNA-seq analysis. *Sci. Rep.* **7**, 43311; doi: 10.1038/srep43311 (2017).

**Publisher's note:** Springer Nature remains neutral with regard to jurisdictional claims in published maps and institutional affiliations.

## Supplementary Material

Supplementary Information

Supplementary Table S2

## Figures and Tables

**Table 1:** Length distribution of assembled transcripts and unigenes.

	Transcripts	Unigenes
**Nucleotides length**		
200–500 bp	19,962	19,172
500–1 kbp	10,486	9,135
1 k–2 kbp	10,976	8,774
>2 kbp	4,908	3,422
Total	46,332	40,503
Minimal length (bp)	224	224
Maximal length (bp)	10,082	10,082
N50 (bp)	1,460	1,386
Median length (bp)	617	540
Average length (bp)	934	873

**Table 2:** List of Full-length CYP450s of *S. dulcis*.

No.	Unigene	Gene	Type	CYP Clan	CYP Family	Accession	Length	Loc^a^
1	TR10211|c0_g1	CYP714E28	non-A	72	CYP714	FX983046	522	S
2	TR10242|c0_g1	CYP98A91	A	71	CYP98	FX983047	513	S
3	TR10539|c0_g1	CYP714G17	non-A	72	CYP714	FX983048	513	S
4	TR10747|c0_g1	CYP92A45	A	71	CYP92	GU592501	505	S
5	TR10864|c0_g2	CYP71CG3	A	71	CYP71	FX983049	505	S
6	TR10966|c1_g2	CYP90B33	non-A	85	CYP90	FX983050	502	S
7	TR11108|c1_g1	CYP94A68	non-A	86	CYP94	FX983051	508	S
8	TR11108|c1_g2	CYP94A69	non-A	86	CYP94	FX983052	512	S
9	TR11115|c0_g1	CYP93B34	A	71	CYP93	FX983053	509	M
10	TR11539|c1_g1	CYP71D491	A	71	CYP71	FX983054	511	S
11	TR12035|c0_g1	CYP71D177	A	71	CYP71	GU205276	504	S
12	TR12047|c1_g1	CYP92B39	A	71	CYP92	FX983055	509	M
13	TR12060|c0_g2	CYP707A134	non-A	85	CYP707	FX983056	466	S
14	TR12152|c0_g1	CYP81Q71	A	71	CYP81	FX983057	503	M
15	TR12152|c0_g2	CYP81Q72	A	71	CYP81	FX983058	493	M
16	TR12184|c0_g2	CYP71D176	A	71	CYP71	GU205275	508	S
17	TR12237|c0_g1	CYP74A1	non-A	74	CYP74	FX983059	537	C
18	TR12688|c1_g1	CYP729A31	non-A	85	CYP729	KR936137	486	S
19	TR12693|c1_g1	CYP72A455	non-A	72	CYP72	FX983060	474	S
20	TR12728|c0_g1	CYP71D492	A	71	CYP71	FX983061	494	S
21	TR12738|c0_g1	CYP80E12	A	71	CYP80	FX983062	502	S
22	TR12781|c0_g1	CYP701A50	A	71	CYP701	KP987567	511	—
23	TR12788|c0_g2	CYP82U12	A	71	CYP82	FX983063	520	S
24	TR12810|c3_g1	CYP76B72	A	71	CYP76	FX983064	491	S
25	TR12810|c3_g3	CYP76B73	A	71	CYP76	FX983065	498	S
26	TR13024|c0_g1	CYP90A44	non-A	85	CYP90	FX983066	478	S
27	TR16200|c0_g1	CYP76S18	A	71	CYP76	FX983067	496	S
28	TR1625|c0_g1	CYP92A46	A	71	CYP92	GU592502	507	S
29	TR1667|c0_g2	CYP85A1	non-A	85	CYP85	FX983068	463	M
30	TR1685|c0_g2	CYP98A92	A	71	CYP98	FX983069	509	M
31	TR17729|c0_g1	CYP73A111	A	71	CYP73	KF306081	505	S
32	TR17864|c0_g1	CYP94D63	non-A	86	CYP94	FX983070	510	S
33	TR17971|c0_g1	CYP704A122	non-A	86	CYP704	FX983071	519	S
34	TR18849|c0_g1	CYP701A51	A	71	CYP701	KP987568	506	S
35	TR19339|c0_g1	CYP74A56	non-A	74	CYP74	FX983072	480	—
36	TR20032|c0_g1	CYP92B40	A	71	CYP92	FX983073	511	S
37	TR20321|c0_g1	CYP710A66	non-A	710	CYP710	FX983074	499	S
38	TR2151|c0_g2	CYP82AP1	A	71	CYP82	FX983075	524	S
39	TR22048|c0_g1	CYP89A145	A	71	CYP89	FX983076	544	S
40	TR24808|c0_g1	CYP728B50	non-A	85	CYP728	FX983077	477	M
41	TR26848|c0_g2	CYP71D490	A	71	CYP71	KT884508	486	S
42	TR2731|c0_g1	CYP716A167	non-A	85	CYP716	FX983078	486	S
43	TR28005|c0_g2	CYP51G1	non-A	51	CYP51	FX983079	496	—
44	TR28584|c0_g1	CYP84A75	A	71	CYP84	FX983080	517	S
45	TR28666|c0_g2	CYP88A71	non-A	85	CYP88	KR936134	495	S
46	TR2880|c0_g2	CYP734A47	non-A	72	CYP734	FX983081	536	S
47	TR2963|c0_g1	CYP72F4	non-A	72	CYP72	FX983082	520	S
48	TR29706|c0_g2	CYP78D7	A	71	CYP78	FX983083	504	S
49	TR29760|c0_g1	CYP71A69	A	71	CYP71	FX983084	523	S
50	TR29820|c0_g1	CYP729A29	non-A	85	CYP729	KR936135	485	S
51	TR30271|c0_g1	CYP71CV2	A	71	CYP71	FX983085	501	S
52	TR3058|c0_g1	CYP81Q76	A	71	CYP81	FX983086	497	S
53	TR3091|c0_g2	CYP81Q77	A	71	CYP81	FX983087	497	S
54	TR31185|c0_g1	CYP81B83	A	71	CYP81	FX983088	504	S
55	TR31301|c0_g1	CYP76S19	A	71	CYP76	FX983089	495	M
56	TR31317|c0_g2	CYP86A123	non-A	86	CYP86	FX983090	520	S
57	TR3662|c0_g1	CYP96A125	non-A	86	CYP96	FX983091	507	S
58	TR3844|c0_g1	CYP76S20	A	71	CYP76	FX983092	498	M
59	TR425|c0_g1	CYP729A30	non-A	85	CYP729	KR936136	485	S
60	TR4741|c0_g1	CYP71D493	A	71	CYP71	FX983093	506	S
61	TR5154|c0_g1	CYP76A45	A	71	CYP76	FX983094	500	M
62	TR5308|c0_g1	CYP94A70	non-A	86	CYP94	FX983095	534	S
63	TR5370|c0_g1	CYP74A57	non-A	74	CYP74	FX983096	473	—
64	TR5460|c0_g1	CYP71D175	A	71	CYP71	GU592503	519	S
65	TR5657|c0_g1	CYP75B104	A	71	CYP75	FX983097	517	M
66	TR5703|c0_g1	CYP704A123	non-A	86	CYP704	FX983098	527	S
67	TR592|c0_g2	CYP749A57	non-A	72	CYP749	FX983099	508	S
68	TR5986|c0_g3	CYP78A185	A	71	CYP78	FX983100	540	S
69	TR5993|c0_g1	CYP749A58	non-A	72	CYP749	FX983101	515	S
70	TR644|c0_g1	CYP71A70	A	71	CYP71	FX983102	514	C
71	TR65|c0_g2	CYP76S22	A	71	CYP76	FX983103	503	S
72	TR6626|c0_g1	CYP71AT136	A	71	CYP71	FX983104	499	S
73	TR6880|c0_g1	CYP81Q73	A	71	CYP81	FX983105	496	M
74	TR6913|c0_g1	CYP72A456	non-A	72	CYP72	FX983106	516	S
75	TR7469|c0_g2	CYP82D128	A	71	CYP82	FX983107	518	S
76	TR7505|c0_g3	CYP72A457	non-A	72	CYP72	FX983108	517	S
77	TR7892|c0_g1	CYP96X6	non-A	86	CYP96	FX983109	516	—
78	TR7907|c0_g1	CYP86A124	non-A	86	CYP86	FX983110	403	M
79	TR7981|c0_g2	CYP76AU1	A	71	CYP76	FX983111	494	M
80	TR8187|c0_g2	CYP707A135	non-A	85	CYP707	FX983112	470	S
81	TR8914|c0_g2	CYP76S21	A	71	CYP76	FX983113	497	M
82	TR9058|c0_g1	CYP704A124	non-A	86	CYP704	FX983114	504	S
83	TR9236|c0_g1	CYP71CV1	A	71	CYP71	FX983115	519	S
84	TR9310|c0_g2	CYP721A50	non-A	72	CYP721	FX983116	504	S
85	TR9381|c0_g1	CYP716D37	non-A	85	CYP716	FX983117	481	S
86	TR9527|c0_g2	CYP716A168	non-A	85	CYP716	FX983118	496	S
87	TR9532|c0_g1	CYP73A148	A	71	CYP73	FX983119	535	S
88	TR9578|c0_g1	CYP81Q74	A	71	CYP81	FX983120	511	S
89	TR9728|c0_g1	CYP81B36	A	71	CYP81	GU592500	502	—
90	TR9811|c1_g1	CYP71D489	A	71	CYP71	KT884507	505	S
91	TR9832|c0_g1	CYP81Q75	A	71	CYP81	FX983121	498	C

^a^Cellular location of the protein predicted by TargetP program. C: Chloroplast, M: Mitochondria, S: Secretory, –: Unknown.

**Table 3:** List of Full-length TPSs of *S. dulcis*.

No.	Unigene	Gene	TPS Family	Putative Function	Accession	Length	Loc^a^
1	TR10305|c0_g1	SdTPS1	TPS-b	monoTPS	FX983122	593	M
2	TR10741|c0_g1	SdTPS2	TPS-b	sesquiTPS	FX983123	543	—
3	TR8397|c0_g2	SdTPS3	TPS-b	monoTPS	FX983124	597	C
4	TR12660|c1_g2	SdTPS5	TPS-b	monoTPS	FX983125	546	—
5	TR5434|c0_g2	SdTPS7	TPS-b	monoTPS	FX983126	590	C
6	TR6053|c0_g1	SdTPS8	TPS-a	sesquiTPS	FX983127	553	—
7	TR9744|c0_g1	SdTPS9	TPS-a	sesquiTPS	FX983128	552	—
8	TR12862|c2_g2	SdTPS10	TPS-a	sesquiTPS	FX983129	569	C
9	TR11869|c1_g1	SdTPS11	TPS-a	sesquiTPS	FX983130	548	—
10	TR3061|c0_g2	SdCPS2	TPS-c	class II diTPS	KP987574	804	C
11	TR12522|c0_g1	SdKSL1	TPS-e/f	class I diTPS	FX983131	804	C
12	TR3279|c0_g2	SdCPS1	TPS-c	class II diTPS	AB169981	825	C
13	TR9171|c1_g2	SdKS	TPS-e/f	class I diTPS	JF781124	790	M

^a^Cellular location of the protein predicted by TargetP program. C: Chloroplast, M: Mitochondria, –: Unknown.
