# Supplementary material for: Elucidation of terpenoid metabolism in Scoparia dulcis by RNA-seq analysis
Source: Sci Rep. 2017 Mar 7;7:43311. doi: 10.1038/srep43311 (PMC5339715; doi:10.1038/srep43311)
Supplement: Supplementary Information [file srep43311-s1.pdf]

Elucidation of terpenoid metabolism in *Scoparia dulcis* by RNA-seq analysis

Yoshimi Yamamura, Fumiya Kurosaki, and Jung-Bum Lee*

Graduate School of Medicine and Pharmaceutical Sciences for Research, University of Toyama, 2630 Sugitani, Toyama, Toyama 930-0194, Japan

* To whom correspondence should be addressed.

Jung-Bum Lee, Ph. D.

Tel: +81-76-434-7580

E-mail: lee@pha.u-toyama.ac.jp

Supplementary Table S1. **Quality of RNA sequencing.**

	Leaf w/o MeJA	Leaf with MeJA	Young leaf	Root	Total
Reads before cleaning	5,777,741	4,174,050	5,409,515	5,287,195	20,648,501
Reads after cleaning	5,742,776	4,135,119	5,359,145	5,222,447	20,459,487
Bases (bp)	864,933,096	622,209,635	807,055,777	785,880,922	3,080,079,430

Supplementary Table S3. Similarity analysis based on the best hit.

Species	Number	Percentage
Sesamum indicum	20,212	65.47
Erythranthe guttata	4,186	13.56
Coffea canephora	801	2.59
Nicotiana sylvestris	524	1.70
Vitis vinifera	507	1.64
Nicotiana tomentosiformis	477	1.55
Solanum tuberosum	264	0.86
Solanum lycopersicum	259	0.84
Theobroma cacao	232	0.75
Citrus sinensis	221	0.72
Others	3.410	11.05

Supplementary Table S4. Abbreviations and accession numbers of terpene synthases

Abberviation	Protein Name	Origin	Accession
AaFS	(*E*)-β-farnesene synthase	*Artemisia annua*	CAC12731
AbCAS	*cis*-abienol synthase	*Abies balsamea*	BAF61135
AbIPS	isopimaradiene synthase	*A. balsamea*	AEL99951
AtECPS	*ent*-copalyl diphosphate synthase	*Arabidopsis thaliana*	AAA53632
AtGLS	(*E,E*)-geranyllinalool synthase	*A. thaliana*	AAO85540
AtKS	*ent*-kaurene synthase	*A. thaliana*	AAC39443
AtTPS10	tricyclene synthase	*A. thaliana*	AAD03382
CaCPS	copalyl diphosphate synthase	*Coffea arabica*	ACQ99373
CbLINS	*S*-linalool synthase	*Clarkia breweri*	AAC49395
CcC8S	copal-8-ol diphosphate synthase	*Cistus creticus* subsp. *creticus*	ADJ93862
CmCPS1	copalyl diphosphate synthase 1	*Cucurbita maxima*	AAD04292
CsCPS	copalyl diphosphate synthase	*Croton sublyratus*	BAA95612
GaDCS	(+)-δ-cadinene synthase	*Gossypium arboreum*	CAA65289
HaCPS1	copalyl diphosphate synthase-like 1	*Hordeum vulgare* var. *distichum*	AAT49065
HaKSL2	*ent*-kaurene synthase like 2	*H. vulgare* var. *distichum*	AAT49067
HaKSL3	*ent*-kaurene synthase like 3	*H. vulgare* var. *distichum*	JAG35806
IeCPS1	copalyl diphosphate synthase	*Isodon eriocalyx*	AEP03177
IeCPS2	copalyl diphosphate synthase	*I. eriocalyx*	AEP03175
LaBES	*exo*-α-bergamotene synthase	*Lavandula angustifolia*	ABB73046
LaLIMS	(*R*)-limonene synthase	*L. angustifolia*	ABB73044
LaLINS	(*R*)-linalool synthase	*L. angustifolia*	ABB73045
LdTPS1	δ-cadinene synthase	*Phyla dulcis*	AFR23368
LdTPS5	bicyclogermacrene synthase	*P. dulcis*	AFR23369
LdTPS6	β-caryophyliene synthase	*P. dulcis*	AFR23370
LdTPS7	*trans*-α-bergamotene synthase	*P. dulcis*	AFR23371
LdTPS8	(+)-*epi*-α-bisabolol synthase	*P. dulcis*	AFR23372
LsECPS1	copalyl diphosphate synthase 1	*Lactuca sativa*	BAB12440
LsKS1	*ent*-kaurene synthase	*L. sativa*	BAB12441
MaLINS	(*R*)-linalool synthase	*Mentha aquatica*	AAL99381
MpFS	β-farnesene synthase	*Mentha* x *piperita*	AEA49040
MrTPS4	(*E*)-β-ocimene synthase	*Matricaria chamomilla* var. *recutita*	AFM43737

MvCPS1	peregrinol diphosphate synthase	*Marrubium vulgare*	AIE77090
MvCPS2	class II diterpene synthase	*M. vulgare*	AIE77091
MvCPS3	(+)-copalyl diphosphate synthase	*M. vulgare*	AIE77092
MvELS	9,13-epoxy-labd-14-en synthase	*M. vulgare*	AIE77094
MvKS	*ent*-kaurene synthase	*M. vulgare*	AIE77093
NsCTS3	cembratrienol synthase 3	*Nicotiana sylvestris*	ADI87448
NtCAS	*cis*-abienol synthase	*N. tabacum*	CCD33019
NtEAS	5-*epi*-aristolochene synthase	*N. tabacum*	AAA19216
NtLPPS	8-hydroxy-copalyl disphosphate synthase	*N. tabacum*	CCD33018
ObCDS	γ-cadinene synthase	*Ocimum basilicum*	AAV63787
ObFES	(-)-*endo*-fenchol synthase	*O. basilicum*	AAV63790
ObGDS	germacrene D synthase	*O. basilicum*	AAV63786
ObGES	geraniol synthase	*O. basilicum*	AAR11765
ObLIS	(*R*)-linalool synthase	*O. basilicum*	AAV63789
ObMYS	β-myrcene synthase	*O. basilicum*	AAV63791
ObSES	selinene synthase	*O. basilicum*	AAV63785
ObTES	terpinolene synthase	*O. basilicum*	AAV63792
ObZIS	α-zingiberene synthase	*O. basilicum*	AAV63788
OsCPS1	*ent*-copalyl diphosphate synthase 1	*Oryza sativa*	BAS78107
OsCPS2	*ent*-copalyl diphosphate synthase 2	*O. sativa*	BAH91759
OsCPS4	*syn*-copalyl diphosphate synthase	*O. sativa*	BAF14085
OsKS1	*ent*-kaurene synthase	*O. sativa*	BAS90962
OsKSL10	*ent*-sandaracopimaradiene synthase	*O. sativa*	BAF29818
OsKSL4	*syn*-pimaradiene synthase	*O. sativa*	BAS87953
OsKSL5	*ent*-pimaradiene synthase	*O. sativa*	BAS79346
OsKSL6	*ent*-kaurene synthase like 6	*O. sativa*	BAS79348
OsKSL7	*ent*-kaurene synthase like 7	*O. sativa*	BAS79338
OsKSL8	stemarene synthase	*O. sativa*	ABA93676
OvTPS2	γ-terpinene synthase	*Origanum vulgare*	ADK73621
OvTPS4	bicyclogermacrene synthase	*O. vulgare*	ADK73618
PaBIS	*E*-α-bisabolene synthase	*Picea abies*	AAS47689
PaLAS	levopimaradiene synthase	*P. abies*	AAS47691

PcGAS	germacrene A synthase	*Pogostemon cablin*	AAS86321
PcGDS	germacrene D synthase 2	*P. cablin*	AAS86320
PcPIS	pimaradiene synthase	*Pinus contorta*	AFU73867
PcTPS17	patchoulol synthase	*Pogostemon cablin*	ABC87816
PcTPSA	γ-curcumene synthase	*P. cablin*	AAS86319
PpCPS/KS	*ent*-kaurene synthase	*Physcomitrella patens* subsp. *patens*	BAF61135
PsECPS	*ent*-copalyl diphosphate synthase	*Picea sitchensis*	ADB55709
PsKS	*ent*-kaurene synthase	*P. sitchensis*	ADB55710
RoCPS1	copalyl diphosphate synthase	*Rosmarinus officinalis*	AHL67261
RoKSL1	kaurene synthase-like 1	*R. officinalis*	AHL67262
SdCPS1	*ent*-copalyl diphosphate synthase	*Scoparia dulcis*	BAD91286
SdKS	*ent*-kaurene synthase	*S. dulcis*	AEF33360
ShSBS	(+)-α-santalene synthase	*Solanum habrochaites*	ACJ38409
SlCPS	copalyl diphosphate synthase	*Solanum lycopersicum*	BAA84918
SmCPS1	copalyl diphosphate synthase 1	*Salvia miltiorrhiza* f. *alba*	AHJ59321
SmCPS2	copalyl diphosphate synthase 2	*S. miltiorrhiza* f. *alba*	AHJ59322
SmCPS3	copalyl diphosphate synthase 3	*S. miltiorrhiza* f. *alba*	AHJ59323
SmCPS4	copal-8-ol diphosphate synthase	*S. miltiorrhiza* f. *alba*	AKN91186
SmCPS5	*ent*-copalyl diphosphate synthase 5	*S. miltiorrhiza* f. *alba*	AHJ59324
SmKS	kaurene synthase	*S. miltiorrhiza*	ABV08817
SmKSL2	kaurene synthase 2	*S. miltiorrhiza* f. *alba*	AHJ59325
SoBS	(+)-bornyl diphosphate synthase	*Salvia officinalis*	AAC26017
SoCS	1,8-cineole synthase	*S. officinalis*	AAC26016
SoSS	(+)-sabinene synthase	*S. officinalis*	AAC26018
SrCPS1	copalyl disphosphate synthase 1	*Stevia rebaudiana*	AAB87091
SrKS1	kaurene syntahse	*S. rebaudiana*	AAD34294
SsLPPS	13-labden-8,15-diol pyrophosphate synthase	*Salvia sclarea*	AED21247
SsSS	sclareol synthase	*S. sclarea*	AFU61898
SsTPS3	terpene synthase 3	*S. sclarea*	AFU61899
TbTXS	taxadiene synthase	*Taxus brevifolia*	AAC49310
VvGLS	(*E,E*)-geranyllinalool synthase	*Vitis vinifera*	ADR74220

Supplementary Table S5. Accession numbers of CYP450s from *Salvia miltiorrhiza*

SmCYP	Accession	SmCYP	Accession	SmCYP	Accession
SmCYP51G1	AJD25147	SmCYP721A38	AJD25252	SmCYP81Q40	AJD25196
SmCYP701A40	AJD25233	SmCYP727B10	AJD25253	SmCYP81Q41	AJD25197
SmCYP704A98	AJD25234	SmCYP728D17	AJD25254	SmCYP81Q42	AJD25198
SmCYP704A99	AJD25235	SmCYP72A326	AJD25167	SmCYP81Q43	AJD25199
SmCYP704B37	AJD25236	SmCYP72A327	AJD25168	SmCYP82D70	AJD25200
SmCYP706C35	AJD25237	SmCYP72A328	AJD25169	SmCYP82D71	AJD25201
SmCYP706G11	AJD25238	SmCYP72A329	AJD25170	SmCYP82U4	AJD25202
SmCYP707A100	AJD25240	SmCYP72A330	AJD25171	SmCYP82V2	AJD25203
SmCYP707A101	AJD25241	SmCYP72A331	AJD25172	SmCYP84A60	AJD25204
SmCYP707A102	AJD25242	SmCYP734A33	AJD25255	SmCYP84A61	AJD25205
SmCYP707A99	AJD25239	SmCYP736A121	AJD25256	SmCYP85A1	AJD25206
SmCYP711A44	AJD25243	SmCYP736A122	AJD25257	SmCYP86A91	AJD25207
SmCYP714A25	AJD25244	SmCYP736A123	AJD25258	SmCYP86A92	AJD25208
SmCYP714E21	AJD25245	SmCYP73A120	AJD25173	SmCYP88A52	AJD25209
SmCYP714G13	AJD25246	SmCYP749A37	AJD25259	SmCYP89A115	AJD25210
SmCYP714G14	AJD25247	SmCYP749A38	AJD25260	SmCYP90A39	AJD25211
SmCYP716A89	AJD25248	SmCYP749A39	AJD25261	SmCYP90B26	AJD25212
SmCYP716C12	AJD25249	SmCYP74A1	AJD25174	SmCYP90C19	AJD25213
SmCYP716D25	AJD25250	SmCYP74B21	AJD25175	SmCYP92A73	AJD25214
SmCYP71A57	AJD25148	SmCYP75A57	AJD25176	SmCYP92B28	AJD25215
SmCYP71A58	AJD25149	SmCYP75B79	AJD25177	SmCYP92B29	AJD25216
SmCYP71A59	AJD25150	SmCYP75B80	AJD25178	SmCYP93B25	AJD25217
SmCYP71AH15	AJD25151	SmCYP76A35	AJD25179	SmCYP94A48	AJD25218
SmCYP71AP14	AJD25152	SmCYP76A36	AJD25180	SmCYP94A49	AJD25219
SmCYP71AT89	AJD25153	SmCYP76AH1	AJD25181	SmCYP94B50	AJD25220
SmCYP71AT90	AJD25154	SmCYP76AK2	AJD25182	SmCYP94C54	AJD25221
SmCYP71AT91	AJD25155	SmCYP76AK3	AJD25183	SmCYP94C55	AJD25222
SmCYP71AT92	AJD25156	SmCYP76G16	AJD25184	SmCYP94D47	AJD25223
SmCYP71AT93	AJD25157	SmCYP76S7	AJD25185	SmCYP96A84	AJD25224
SmCYP71AU51	AJD25158	SmCYP76T27	AJD25186	SmCYP96A85	AJD25225
SmCYP71AU52	AJD25159	SmCYP77A27	AJD25187	SmCYP97A41	AJD25226

SmCYP71AU53	AJD25160	SmCYP77A28	AJD25188	SmCYP97B34	AJD25227
SmCYP71BE37	AJD25161	SmCYP78A113	AJD25189	SmCYP97C28	AJD25228
SmCYP71D374	AJD25162	SmCYP78A114	AJD25190	SmCYP98A75	AJD25229
SmCYP71D410	AJD25163	SmCYP78A115	AJD25191	SmCYP98A76	ACA64047
SmCYP71D411	AJD25164	SmCYP79D40	AJD25192	SmCYP98A77	AJD25231
SmCYP71D412	AJD25165	SmCYP81B61	AJD25193	SmCYP98A78	AJD25232
SmCYP71D413	AJD25166	SmCYP81B62	AJD25194		
SmCYP720A1	AJD25251	SmCYP81C16	AJD25195		

Supplementary Table S6. Accession numbers of CYP450s from *Arabidopsis thaliana* and their function

AtCYP	Accession	Function
AtCYP51G1	BAB61873	sterol 14-demethylase
AtCYP71A13	AAC02748	indoleacetaldoxime dehydratase
AtCYP71B15	BAB01230	bifunctional dihydrocamelexate synthase/camalexin synthase
AtCYP72C1	AAD50024	6-deoxycastasterone oxidase
AtCYP73A5	AAB58356	*trans*-cinnamate 4-monooxygenase
AtCYP74B2	AAC69871	linolenate hydroperoxide lyase
AtCYP75B1	AAG16746	flavonoid 3'-monooxygenase
AtCYP79A2	AAF70255	phenylalanine *N*-monooxygenase
AtCYP79F1	AAD34693	dihomomethionine *N*-hydroxylase
AtCYP81F2	CAR63887	indole glucosinolate hydroxylase
AtCYP82G1	BAB02077	nerolido/geranyllinalool convert to C-11/C-16 homoterpenes
AtCYP83B1	BAA28531	indole-3-acetooxime oxidase
AtCYP85A1	BAB60858	brassinosteroid 6-oxidase
AtCYP86A1	CAA62082	fatty acidomega-hydroxylase
AtCYP88A3	AAK11564	*ent*-kaurenoic acid oxidase
AtCYP90B1	AAC05093	6-deoxocathasterone C-22 hydroxylase
AtCYP90C1	BAA37161	33-*epi*-6-deoxyocathasterone 23-monooxygenase
AtCYP90D1	BAB62109	3-*epi*-6-deoxyocathasterone 23-monooxygenase
AtCYP96A15	AAG50737	alkane hydroxylase
AtCYP97C1	AAR83120	carotene epsilon-monooxygenase
AtCYP98A3	AAB86449	phenolic ester 3'-hydroxylase
AtCYP701A3	AAC39507	*ent*-kaurene oxidase
AtCYP703A2	AAF97323	medium-chain saturated fatty acid hydroxylase
AtCYP707A1	BAD16629	abscisic acid 8'-hydroxylase
AtCYP708A2	BAB11064	thalianol hydroxylase
AtCYP710A1	BAE71351	sterol C-22 desaturase
AtCYP711A1	AAC14532	cariactone oxidase
AtCYP714A1	AAL24168	GA12 oxidase
AtCYP734A1	AAB95305	brassinolide C-26 hydroxylase
AtCYP735A1	BAB09357	cytokinin hydroxylase

Supplementary Table S7. List of primers used for qRT-PCR

Primers	5' – 3'
SdCYP4-FW	AGATGCTCAAGGCGATGAGTTG
SdCYP4-RV	ATCCAATTTCTCCGCCCGATTC
SdCYP7-FW	TCACCTTTGCATACAGGCCTTC
SdCYP7-RV	TGCGCACTTGTCTCCAGTATTC
SdCYP17-FW	AAGATGCAGAGAGCTTCATGCC
SdCYP17-RV	AAAGACTTACCGGGGCAAATCC
SdCYP27-FW	TTGTTGCGGGAACAGATACGAC
SdCYP27-RV	ATCCGCTTCGTCTATCGCTTTC
SdCYP28-FW	ATCCTAAACGTACGCAGCCAAG
SdCYP28-RV	TGAGCTGAAAAGGCTGGAGTTG
SdCYP30-FW	TGCTGGAATGTTGCCTAGCAG
SdCYP30-RV	AGTCATACCCCGCAAAGTCAAC
SdCYP49-FW	AGAGAAGCCATGGAGAATTGCG
SdCYP49-RV	TATTTTGGGTCCCTTCCAAGCG
SdCYP56-FW	AGCTGCACGAATATGTGAGTGG
SdCYP56-RV	ATCTCCTGCAACGAATCCGAAC
SdCYP57-FW	TCGTCCTCGCATTTTGGCTATC
SdCYP57-RV	AATAATGAGCGGCAAGGAGGTG
SdCYP66-FW	AGGCTGCAGAACGAAATGAGAG
SdCYP66-RV	AATGGAATGGGCACGTGTAGTC
SdCYP77-FW	TGGTGGAGGCTTTGCTTGATC
SdCYP77-RV	TGGCGTATCAACTCCGTCATTG
SdCYP79-FW	AAGCTGCTCCATCTGTTCAGTG
SdCYP79-RV	CATTCATGGCAGCTTGCTTGTG
UnivF-1131	AAACTTAAAGGAATTGACGG
18SrRNA-RV	GAAGGGATACCTCCGCATA

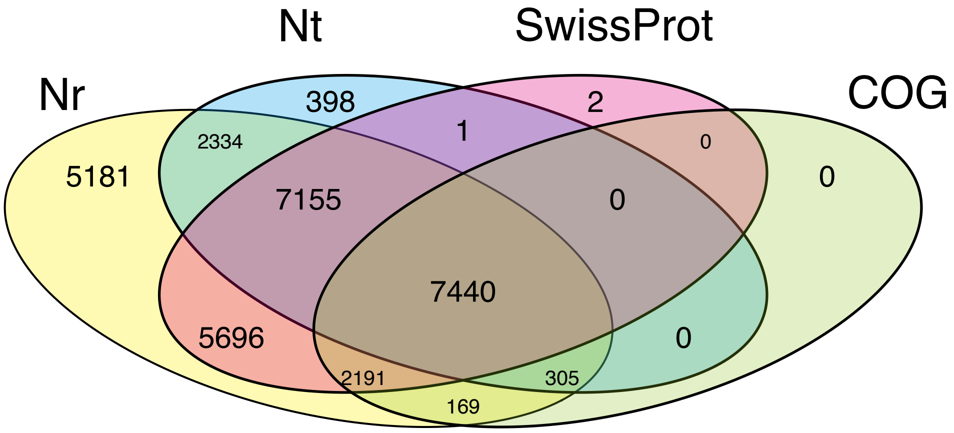

Supplemental Figure S1. Venn diagram indicating annotated genes by the Nr, Nt, SwissProt, and COG databases. The numbers of genes annotated is listed in each diagram component.

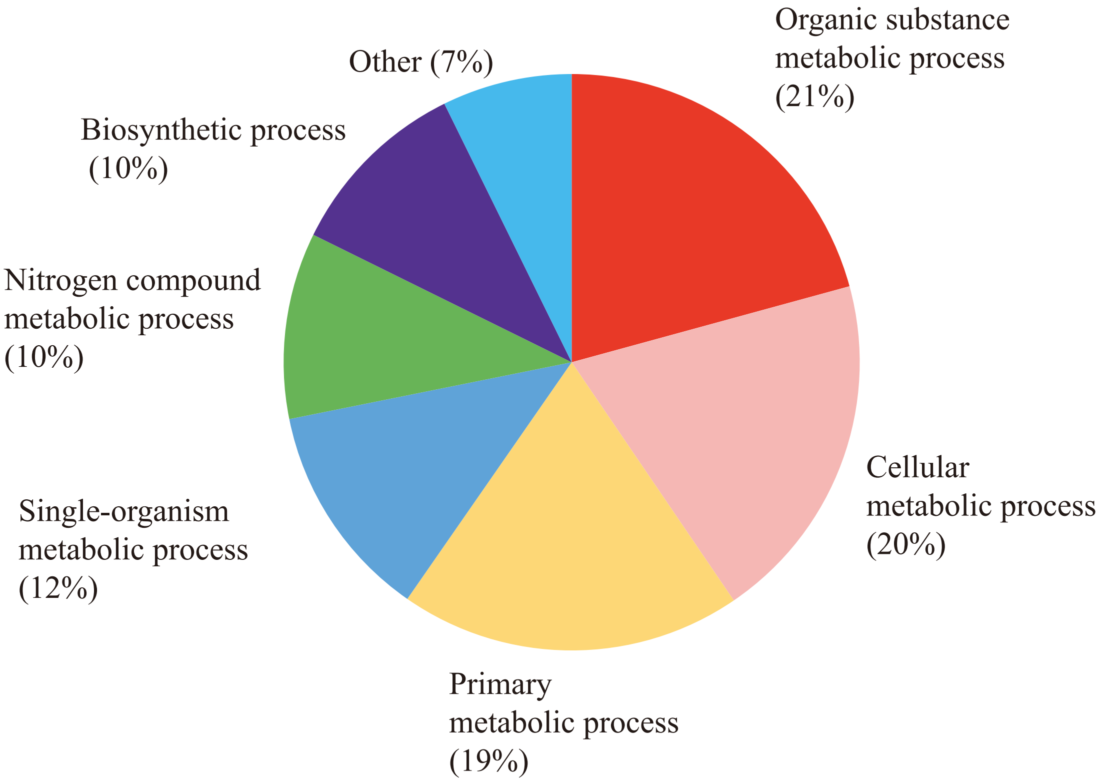

Supplemental Figure S2. Gene classification by metabolism of metabolic process in Figure 1.

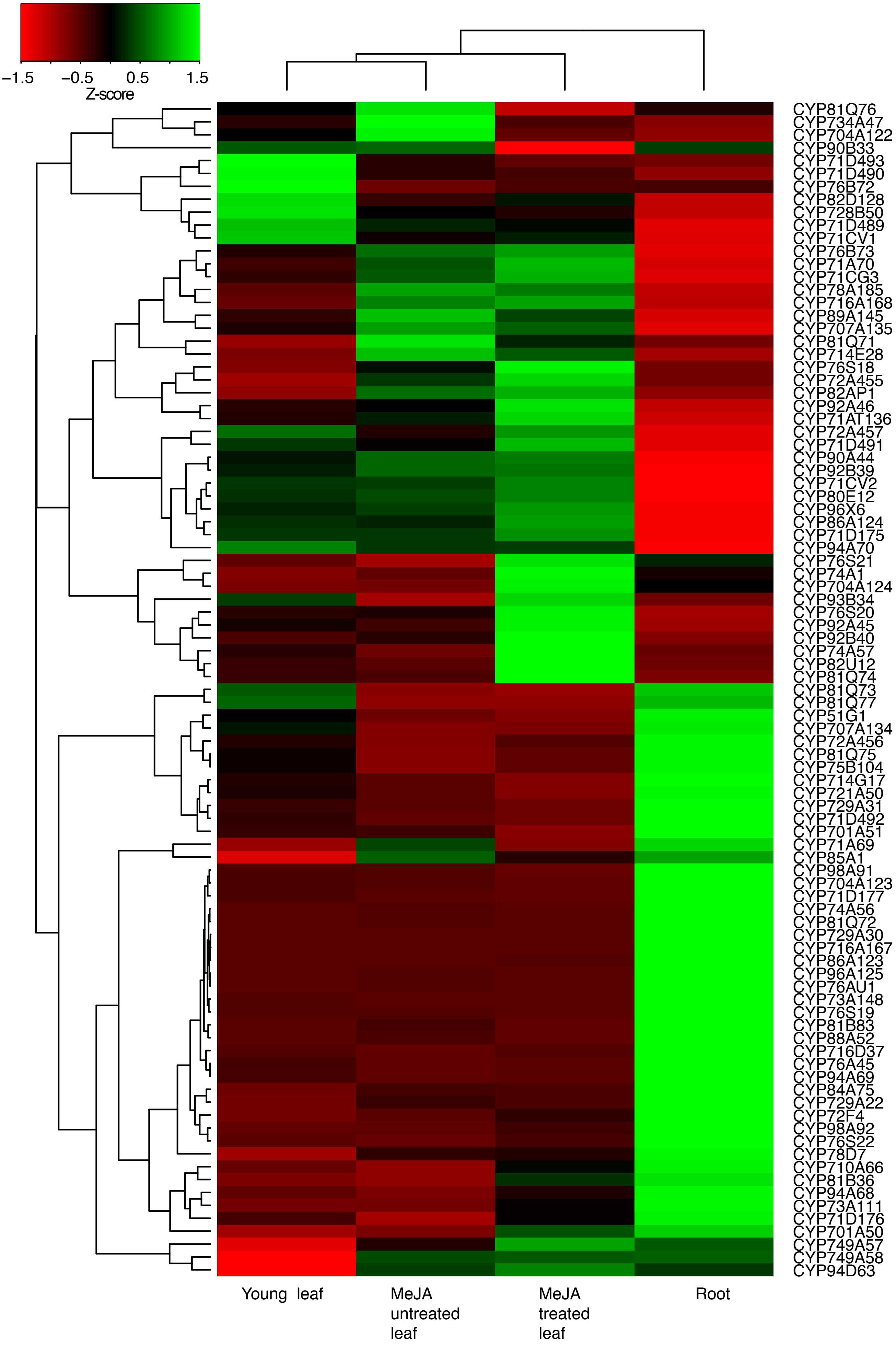

Supplemental Figure S3. Hierarchical clustering analysis of SdCYP genes expression profiles from 4 tissues. Color gradient illustrated the Z-scores of the gene expression values by calculating as FPKM values.

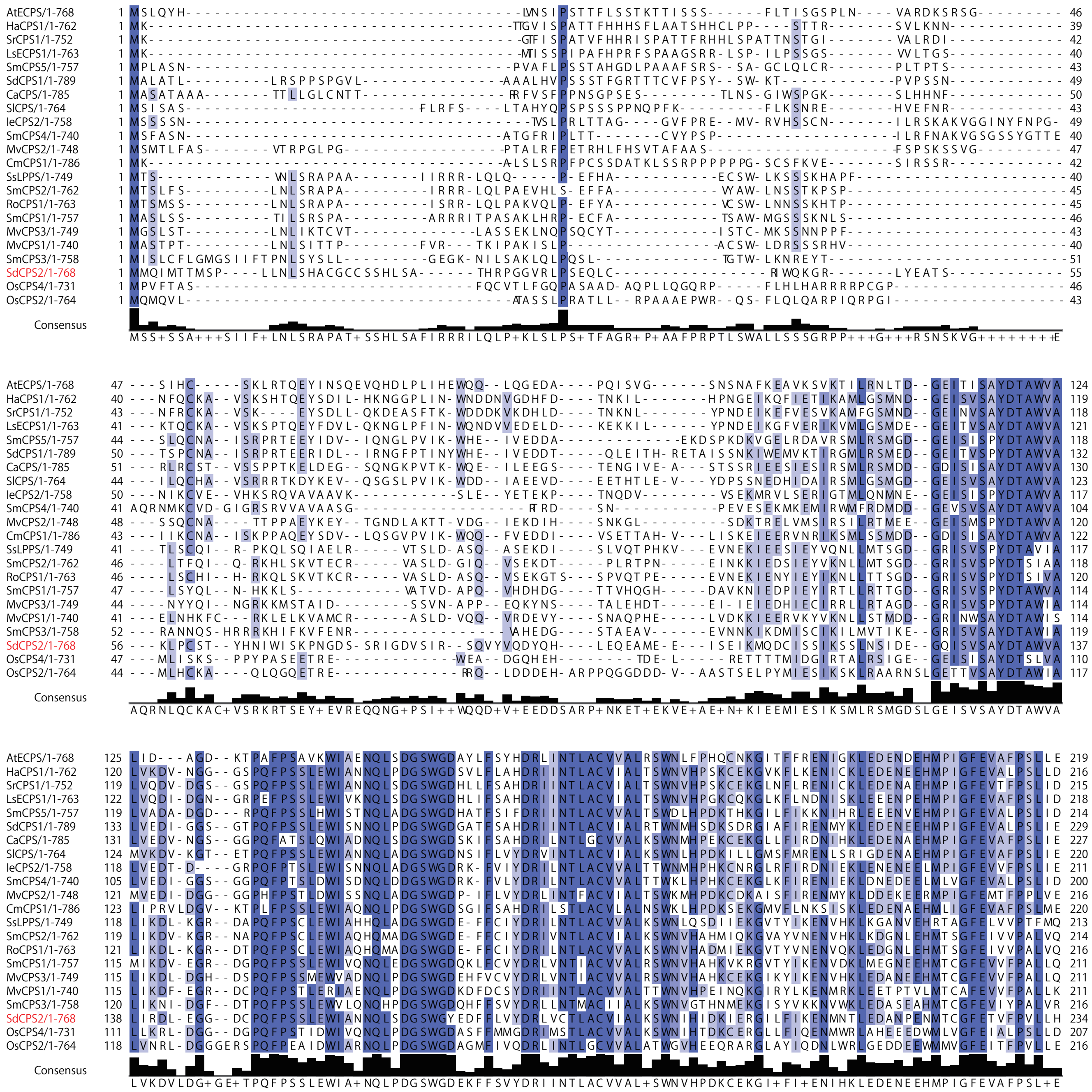

Supplementary Figure S4

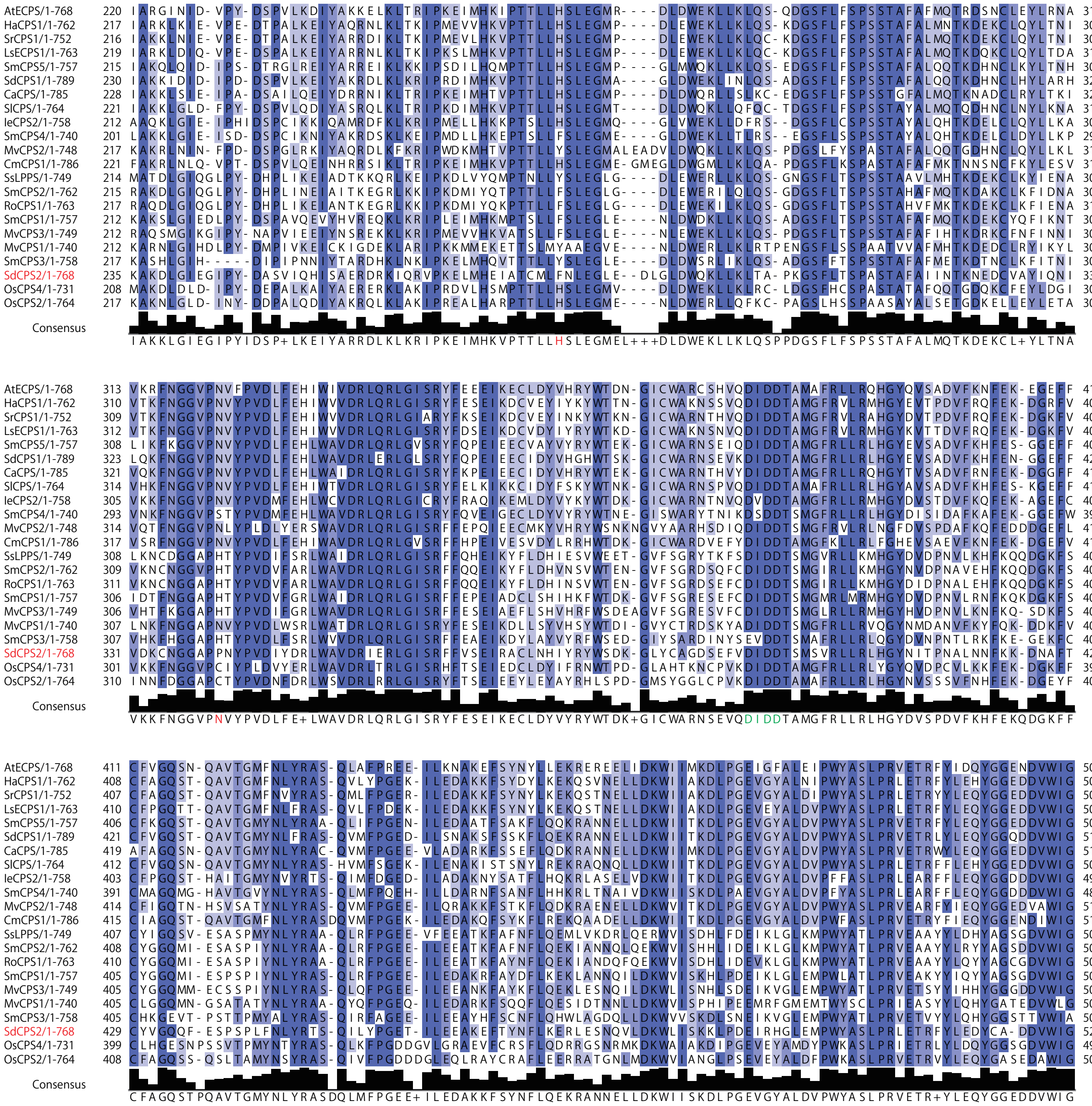

Supplementary Figure S4 (continued).

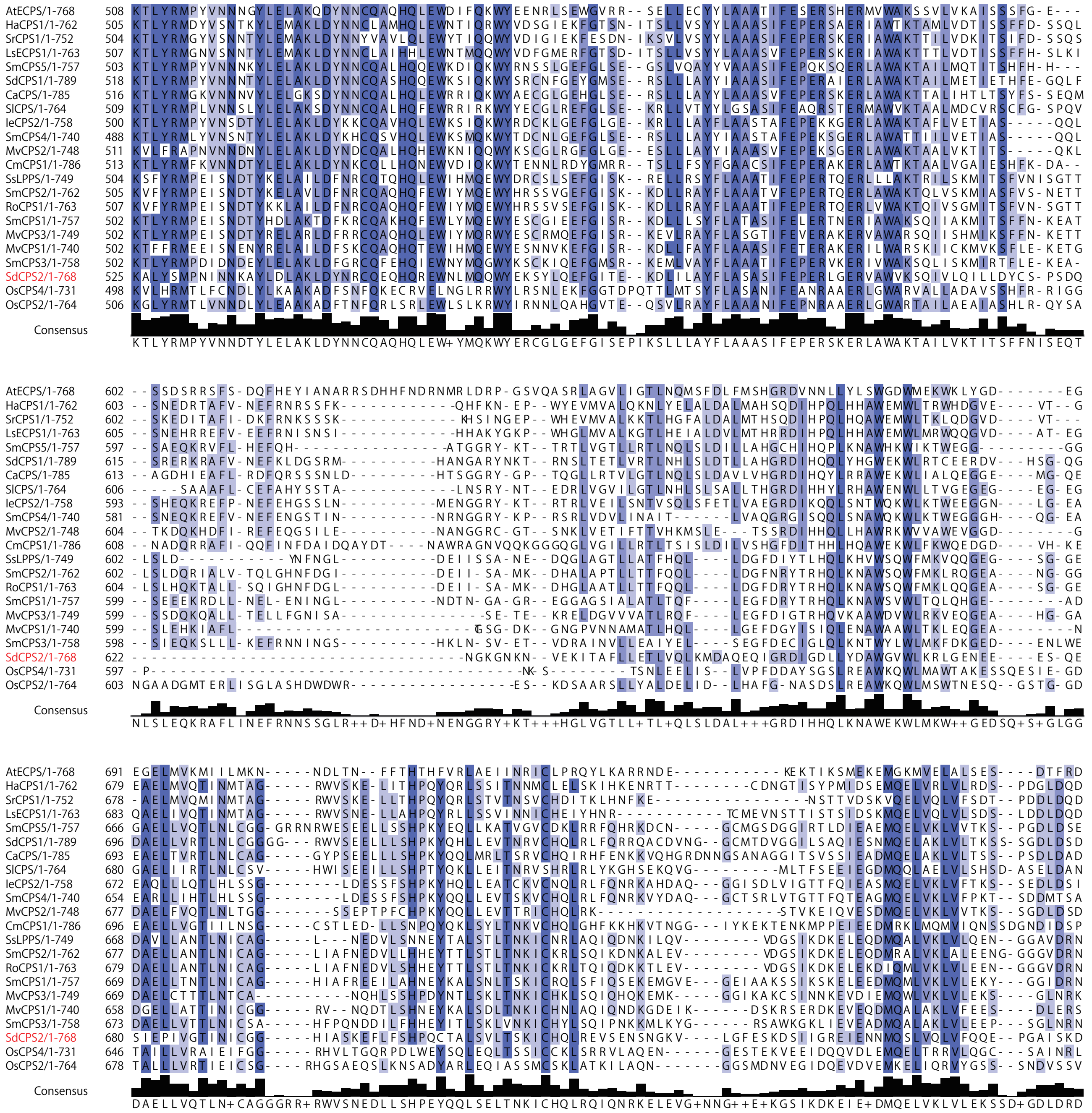

Supplementary Figure S4. Alignment of the amino acid sequences of class II diterpene cyclases. Annotaions: “DxDD” motif is green, key catalytic base dyad is red.

Full names of the sequences are shown in Supplementary Table S4.
